# Disease Recurrence during Adjuvant Immune Checkpoint Inhibitor Treatment in Metastatic Melanoma: Clinical, Laboratory, and Radiological Characteristics in Patients from a Single Tertiary Referral Center

**DOI:** 10.3390/ijms231810723

**Published:** 2022-09-14

**Authors:** Jonas K. Kurzhals, Gina Klee, Victoria Hagelstein, Detlef Zillikens, Patrick Terheyden, Ewan A. Langan

**Affiliations:** 1 Department of Dermatology, University of Lübeck, 23552 Lübeck, Germany; 2 Dermatological Sciences, University of Manchester, Manchester M13 9PR, UK

**Keywords:** immune checkpoint, melanoma, adjuvant therapy, disease recurrence

## Abstract

Despite the dramatic improvements in recurrence-free survival in patients with metastatic melanoma treated with immune checkpoint inhibitors (ICI), a number of patients develop metastases during adjuvant therapy. It is not currently possible to predict which patients are most likely to develop disease recurrence due to a lack of reliable biomarkers. Thus, we retrospectively analyzed the case records of all patients who commenced adjuvant ICI therapy between January 2018 and December 2021 in a single university skin cancer center (*n* = 46) (i) to determine the rates of disease recurrence, (ii) to examine the utility of established markers, and (iii) to examine whether re-challenge with immunotherapy resulted in clinical response. Twelve out of forty-six (26%) patients developed a relapse on adjuvant immunotherapy in our cohort, and the median time to relapse was 139 days. Adjuvant immunotherapy was continued in three patients. Of the twelve patients who developed recurrence during adjuvant immunotherapy, seven had further disease recurrence within the observation period, with a median time of 112 days after the first progress. There was no significant difference comparing early recurrence (<180 days after initiation) on adjuvant immunotherapy to late recurrence (>180 days after initiation) on adjuvant immunotherapy. Classical tumor markers, including serum lactate dehydrogenase (LDH) and S-100, were unreliable for the detection of disease recurrence. Baseline lymphocyte and eosinophil counts and those during immunotherapy were not associated with disease recurrence. Interestingly, patients with NRAS mutations were disproportionately represented (60%) in the patients who developed disease recurrence, suggesting that these patients should be closely monitored during adjuvant therapy.

## 1. Introduction

Disease outcomes for patients with metastatic and locally advanced melanoma and non-melanoma skin cancer have been dramatically improved by the use of systemic immuno- and targeted therapy. Especially, immune checkpoint inhibitors targeting the programmed cell death (PD-1)-programmed cell death ligand (PD-L1) axis, as well as those targeting the cytotoxic T-lymphocyte antigen 4 (CTLA-4) and the lymphocyte-activation gene 3 (LAG-3), now play key roles in the management of a range of advanced skin cancers including melanoma, Merkel cell carcinoma, basal cell carcinoma, and squamous cell carcinoma [1,2,3,4,5,6,7]. Moreover, there is an increasing tendency to employ immune checkpoint inhibitors earlier in the disease process, with pembrolizumab (anti-PD-1) now licensed for completely resected melanoma from stage IIB/C, i.e., in the absence of metastatic disease [8].

Despite these treatment advances, the incidence of malignant melanoma continues to rise, especially in fair-skinned individuals. With more than 132,000 cases annually, melanoma is already the fifth most common cancer in the USA [9,10]. Treatment is multi-modal, but surgical excision, with adequate resection margins, is the mainstay of initial therapy. Depending on disease classification and sentinel lymph node status, checkpoint inhibitor-based immunotherapy plays a key role in further management. Targeted therapy with B-Raf (BRAF) and mitogen-activated protein kinase (MEK) inhibitors provides an additional treatment option in patients with a BRAFV600 mutation; now licensed in both adjuvant and palliative settings [11]. Stereotactic radiotherapy also plays an important role in the management of patients with symptomatic melanoma brain metastases [12].

Nevertheless, immune checkpoint inhibitor therapy has revolutionized the treatment for both locally advanced and metastatic melanoma by targeting the cytotoxic T-lymphocyte antigen 4 (CTLA-4) and the programmed cell death 1 (PD1) proteins [6,13]. Treatment with immune checkpoint inhibition has also been licensed in adjuvant settings to prevent tumor recurrence in patients with stage IIB, IIC, III, and IV diseases, who are clinically and radiologically tumor-free after surgery. The CheckMate 238 and Keynote-054 clinical trials both demonstrated significantly longer recurrence-free survival for patients treated with immunotherapy in this setting [14,15] The Keynote-054 reported a distant free survival of 65.3% at 3.5 years (median follow-up 42.3 months) in patients treated with pembrolizumab every three weeks (200 mg) compared to 49.4% in the placebo group (*p* < 0.0001) [16]. Moreover, recurrence-free survival in the pembrolizumab group was 59.8% vs. 41.4% in the placebo group at 3.5 years of follow-up. The CheckMate 238 trial compared the use of nivolumab (3 mg/kg every two weeks) versus ipilimumab (10 mg/kg every three weeks) in resected stage IIIB-C and stage IV melanoma. The four-year recurrence-free survival was 51.7% in the nivolumab group and 41.7% in the ipilimumab group (*p* = 0.0003) [15].

However, a sizable number of patients failed to respond to immune checkpoint inhibitor therapy due to innate resistance or lost response over time (acquired resistance) [17]. Up to 60% of patients showed innate resistance to immune checkpoint inhibitors, and almost one-third of initial responders acquired resistance in the palliative treatment setting [18]. Following adjuvant immunotherapy, up to 30% of the patients develop disease recurrence within one year [19]. Due to a lack of reliable baseline biomarkers, it is currently not possible to accurately identify which patients are most likely to develop disease recurrence during adjuvant therapy.

The value of measuring serum S-100 concentrations as a quantitative biomarker in the routine follow-up of patients with high-risk melanoma is well-established [20]. In fact, both serum S-100 and LDH measurements are prognostic markers for patients with metastatic melanoma treated with immune checkpoint therapy. For example, Wagner et al. illustrated that patients with unresectable melanoma (stage III or stage IV) with elevated S-100 and LDH concentrations at baseline had an impaired overall survival (OS) compared to patients whose serum S-100 and LDH concentrations were within normal limits. This was the case not only for patients receiving anti-PD1 monotherapy but also for those who underwent treatment with combined immunotherapy. Wagner et al. also showed that patients with elevated baseline S-100/LDH concentrations being treated with pembrolizumab had a significantly lower one-year overall survival compared to patients with normal serum S-100 concentrations (51.1% vs. 83.1%) and normal LDH concentrations (44.4% vs. 80.8%) [21]. Diem et al. confirmed this finding, reporting that patients being treated with both nivolumab and pembrolizumab had significantly shorter overall survival if they presented with elevated baseline LDH before the initiation of immunotherapy [22]. Moreover, increased absolute or relative lymphocyte and eosinophil counts at baseline and during immunotherapy are strongly associated with disease control and survival [23,24].

However, the utility of these markers, both in the real-world setting and in the context of adjuvant immunotherapy, is currently unclear. Indeed, given the increasing use of adjuvant immunotherapy across a range of cancer entities, there is a pressing need for real-world data in this patient group [19].

Therefore, we report the clinical and laboratory characteristics of patients who underwent immunotherapy between 1 January 2018 and 31 December 2021, in the Department of Dermato-Oncology at the University Hospital in Lübeck, Germany. We sought to identify the rates of disease recurrence and to examine the utility of established markers of response to immunotherapy in the palliative setting. Moreover, in addition to recording the site and the extent of disease recurrence, we aimed to examine whether re-challenge with immunotherapy resulted in clinical response. 

## 2. Results

### 2.1. Characteristics

During January 2018 and December 2021, 12 out of 46 patients (26%) suffered a melanoma recurrence during one year of adjuvant immunotherapy treatment. Six of those patients were males, and six were females. The mean age of the patients was 62 years (+/− 9). Three patients suffered from melanoma stage IIIB, six suffered from melanoma stage IIIC, and three suffered from melanoma stage IV. Seven of those patients were treated with nivolumab, and five were treated with pembrolizumab. All patients had cutaneous melanoma. No patient with mucosal melanoma was part of the study. 

### 2.2. Time to Recurrence and Overall Survival

**Figure 1:**
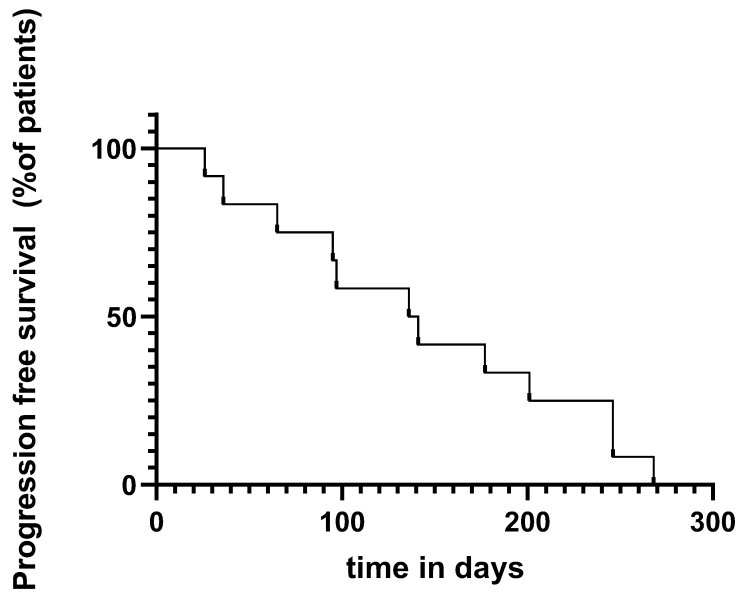
Progression-free survival (all patients).

**Figure 2:**
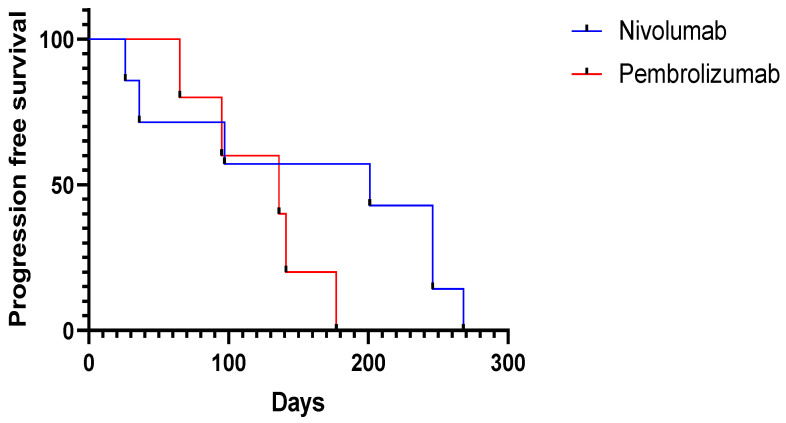
Progression-free survival (nivolumab vs. pembrolizumab) did not illustrate a significant difference (*p* = 0.17).

**Figure 3:**
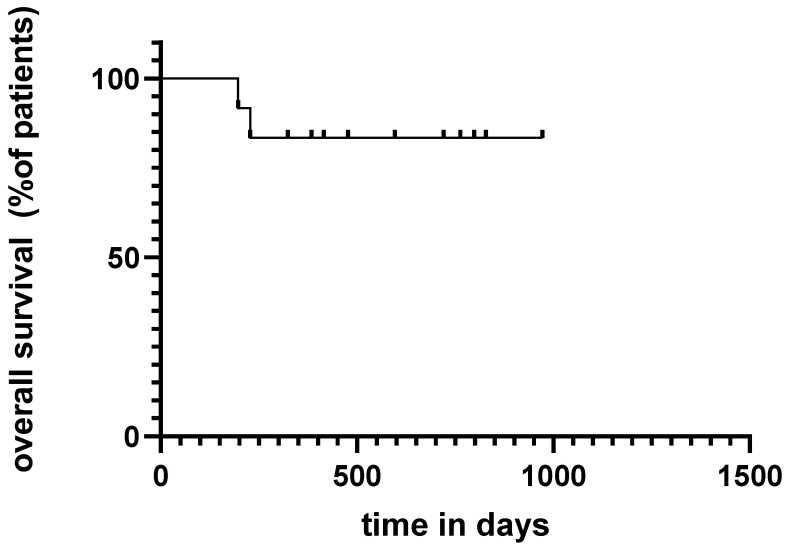
Overall survival of the patients: two patients died within the one-year adjuvant immunotherapy.

The median time of developing recurrence was 139 days (4.6 months) (range: 26–268 days) (Figure 1). For adjuvant nivolumab treatment, the median recurrence time was 201 days (range: 26–268 days) during adjuvant nivolumab treatment compared with 136 days (range: 65–177) during pembrolizumab therapy. There was no statistically significant difference between the groups in terms of median time to recurrence (*p* = 0.17) (Figure 2). Moreover, there was no statistically significant difference comparing stage IIIB and stage IIIC melanoma based on early progress (<180 days) to “late” progress (>180 days) after the initiation of treatment using the Fisher test (*p* = 0.9). In addition, we could not observe a statistically significant difference comparing stage III and stage IV melanoma patients based on early progress and “late” progress, using the Fisher test (*p* = 0.2). Of the nine stage III melanoma patients, the vast majority (*n* = 7, 78%) developed locoregional metastases. Only two of the stage III (22%) melanoma patients developed distant metastases (both lung metastases). One of those two was suffering from an immunosuppressive underlying disease (rheumatoid arthritis) and was undergoing treatment with methotrexate. Of the 12 patients, two patients died within the one-year period of adjuvant immunotherapy, one due to immune-related adverse events only (immune-related colitis stage 4) and the other one due to both adverse events (rhabdomyolysis) and disease progression (Figure 3). 

### 2.3. Management after Recurrence

**Figure 4:**
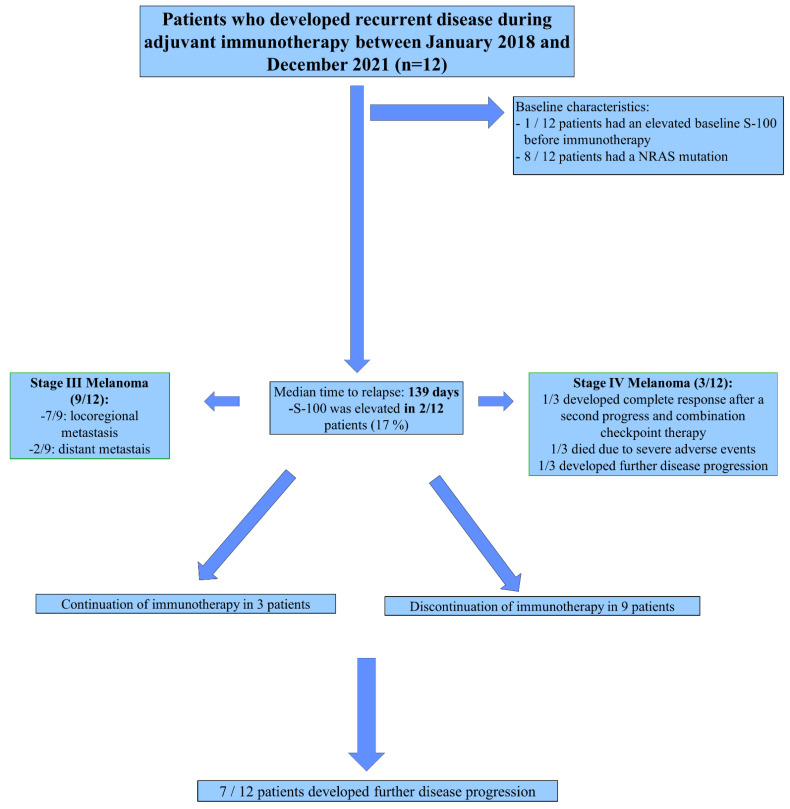
Flowchart overview.

Recurrence during adjuvant treatment was detected by regular clinical examination and carried out prior to each administration of immunotherapy in four of the twelve patients (33%), i.e., before the standard three monthly follow-up examinations that are recommended by the German national melanoma guidelines [25]. In eight of the twelve patients (66%), progress was detected during the recommended follow-ups and staging examinations, as suggested in these guidelines. Of the nine patients who developed locoregional recurrence (78%), all underwent complete resection of metastases (Table 1). Therefore, none of the patients required additional treatment with radiotherapy. Adjuvant anti-PD-1 treatment was re-started in three patients (33%) (Table 1). Of the three stage IV melanoma patients who developed progress, one patient received additional immune checkpoint combination therapy with ipilimumab (anti-CTLA-4) and nivolumab (anti-PD-1) and developed a complete response until the end of the observation period (31 December 2021). The two other patients, who progressed to stage IV, were unable to continue with immunotherapy due to severe adverse events (CTCAE grade 4). One of those patients died, as described above, and the other one continued the therapy with dacarbazine but developed progress again several months later. Seven of the twelve patients suffered additional tumor progress with a median time of 112 days after detection of the first progress (range: 30–887 days) (Figure 4: Flowchart). 

### 2.4. Systemic Biofactors and Mutation Status at Baseline and at the Time of Recurrence 

Of all the patients who had recurrent disease, none had an elevated serum lactate dehydrogenase (LDH) at baseline before starting the adjuvant immunotherapy, which is known to be associated with a significant reduction in overall survival [21,22]. In addition, the patients did not present with higher relative lymphocyte counts at baseline, which is also known to be associated with disease control [24]. The mean baseline percentage was 24% (+/−7). 

Moreover, both baseline eosinophil (normal range: 0.02–0.5 × 10^9^) count and the increase during immunotherapy are known to correlate with disease control and overall survival in patients with non-resectable melanoma [26,27]. The mean baseline eosinophil count was 0.23 (+/−0.2). An increase in the eosinophil count only appeared in two patients, but immunotherapy was not re-started after progress in those. 

In addition to that, we analyzed whether the S-100 level was elevated at both baselines and at the point of progress, which is known to demonstrate tumor growth and progressive disease [24,28,29]. Only 1 of the 12 patients had an elevated baseline S-100 level before starting the adjuvant immunotherapy. The S-100 level was only elevated in two patients at the point of progress (17%), which at least made this tumor marker in our patient cohort an unreliable marker concerning progress. Interestingly, the patient with the elevated baseline S-100 level developed a normal S-100 at the point of progress.

Interestingly, 8 of the 12 patients whose disease recurred during the one-year adjuvant immunotherapy in our department had an NRAS mutation (67%), which is more than three times higher than the normal incidence of NRAS mutation in melanoma patients (normal incidence: 15–20%) and known to be associated with a poorer outcome in immunotherapy [30,31].

### 2.5. Additional Investigations

There was no evidence that sex impacted the risk of disease recurrence in our cohort, with equal numbers of males and females being affected (Table 2). We specifically examined the effect of sex given evidence that females may respond less well to immune checkpoint-based immunotherapy [32]. The overwhelming majority of our patients were overweight, with only one patient with a body mass index (BMI) of <24 suffering from disease recurrence. Eight of the patients had a BMI between 25 and 30, and three patients had a BMI > 30 (Table 2). Whilst this was a small number of patients, there was no indication that overweight patients had a better response to immunotherapy, as has been reported in patients treated in the palliative setting [33,34,35]. The development of immune-related adverse events has also been shown to correlate with treatment response in advanced melanoma [36,37,38]. Only two of the twelve patients in our cohort developed immune-related adverse events during the therapy; ten patients did not. These findings are consistent with the rate of grade III–IV adverse events in the pembrolizumab treatment arm of the Keynote-716 trial (pembrolizumab versus placebo as adjuvant therapy in completely resected stage IIB or IIC melanoma) [8].

Additionally, we analyzed the baseline neutrophil-to-lymphocyte ratio in our patient cohort, since an elevated ratio is associated with poorer outcomes to immunotherapy [39]. Of the twelve patients, only four of the patients had an elevated ratio at baseline. Finally, given the potential of concomitant antibiotic therapy to negatively impact the efficacy of immunotherapy, we examined whether our patients had received antibiotic therapy immediately prior to the initiation of immune checkpoint inhibition [40]. However, none of our patients whose disease had recurred during adjuvant immunotherapy had been prescribed antibiotics prior to the initiation of treatment (Table 2). 

## 3. Discussion

Selecting the best management strategy for melanoma patients who progressed during adjuvant immunotherapy remains a challenge. Combined treatment with CTLA-4 (ipilimumab) and PD-1 (nivolumab) inhibition or BRAF/MEKi, if the tumor shows the specific mutation, are among the only useful options for patients who recur on adjuvant therapy [41,42,43,44]. However, advances in our understanding of melanoma pathogenesis, combined with new insights into tumor immunobiology and the microenvironment, mean that novel therapies including combinatorial immunotherapy, personalized vaccines, small molecules, and manipulating the gastrointestinal microbiome may yield yet more efficacious and safe therapeutic options [45,46,47].

At present, the treatment options for patients lacking a BRAF V600E/K mutation [48] who develop disease recurrence during adjuvant immunotherapy are limited. It has been suggested that PD-1 resistance is not necessarily associated with CTLA-4 resistance and that ipilimumab activity (anti-CTLA-4) is comparable to patients in PD-1-naïve and PD-1-progressive patients, which gives at least the patients who develop distance metastases another treatment option [19,44]. For the patients who progressed on adjuvant immunotherapy with a BRAF V600E/K mutation, BRAF/MEKi is an additional and therapeutic option, even though the durable survival rate is low and can only be achieved in few patients [19,48].

Here, we gathered real-world data from patients who developed tumor recurrence during or after adjuvant immunotherapy at the Department of Dermato-Oncology at the University Hospital in Lübeck, Germany. The median time for the development of progress in our cohort was 139 days (range: 26–268 days). The German melanoma guidelines [25] recommended radiological imaging every six months during adjuvant therapy. Based on our experience, more frequent imaging, particularly of the loco-regional area and in patients with NRAS mutations, should be considered.

In addition to that, we could not find a significant difference concerning progression-free survival by comparing the two different immunotherapies available: nivolumab and pembrolizumab (Figure 2) (*p* = 0.17). Nevertheless, we demonstrated that progress in 2 of the 12 patients (17%) occurred within the first forty days of treatment, and we illustrated that, in four of the patients, the progress was detected during additional clinical examinations prior to each administration of immunotherapy. Therefore, we recommend a thorough clinical examination before each treatment cycle. This is particularly important given the different cycle lengths (nivolumab: q2w and q4w) and pembrolizumab (q3w and q6w). It may be prudent to commence treatment with the shorter cycle (q2w or q3w) to enable more frequent clinical examination, in order to facilitate detect early detection of disease recurrence and, where indicated, perform lymph node sonography. In addition to that, patients should also be informed about the possibility of adverse events reportedly associated with cycle length, such as diabetes mellitus [49]. Moreover, there was no significant difference comparing early and late recurrence during adjuvant immunotherapy. 

The optimal treatment strategy following disease recurrence is unclear and likely depends on whether the patient has developed the loco-regional or distant disease. Of the 12 patients who developed progress, nine developed a locoregional recurrence alone and underwent surgery without adjuvant radiation. It still is a matter of debate as to whether adjuvant immunotherapy should be restarted after locoregional surgery. In our cohort, adjuvant immunotherapy was re-started again in three patients, of whom two remain without recurrence until today and one developed recurrence 887 days after the first progress. Adjuvant immunotherapy was not recommenced in the other six patients. These patients were treated with surgery after locoregional or resectable recurrence based on individual decisions of both our interdisciplinary tumor board and patient choice. Three of the six patients remained recurrence-free until 31 December 2021, whilst three developed further disease manifestations (median time of 102 days, range: 30–154). 

An elevated serum lactate dehydrogenase is known to be an independent baseline biomarker concerning overall survival in patients receiving immune checkpoint inhibitor therapy compared to patients with normal lactate dehydrogenase [21,22,50]. Based on our real-world data, we were unable to illustrate this in our patient cohort receiving adjuvant immunotherapy. None of the patients presented with an elevated baseline LDH. It might be the case that most studies on LDH as a reliable marker were made in patients suffering from advanced melanoma stage IV in the palliative setting and not in the adjuvant setting [24,50]. Nevertheless, we were able to clearly illustrate that patients with a normal LDH at baseline showed disease recurrence. 

Concerning circulating lymphocytes and eosinophil count, patients with recurrence did not present with an elevated baseline count, nor did they develop an increase during the therapy, which has been described to be associated with disease control and overall survival [24,26,27,29]. Therefore, patients whose lymphocyte and eosinophil counts do not increase during immunotherapy should be closely monitored for disease recurrence.

An important aspect of our study is that the calcium-binding protein S-100 was an unreliable marker for disease recurrence in our patient cohort. Serum S-100 concentration is a recognized prognostic marker at baseline but is also useful for detecting melanoma progression [24,51,52]. Only one patient had an elevated S-100 at baseline, and only two of the patients showed an increase at the point of progress. This leads to the question of whether this marker can and should be used during adjuvant immunotherapy to evaluate treatment response or tumor progress. Further multicentric analyses are necessary to definitely answer this question. 

We were able to illustrate that, in our patient cohort, nine out of twelve patients (75%) presented with an NRAS mutation, which normally only occurs in about 15–20% of melanomas, is known to be more aggressive, and is associated with poorer outcomes [53]. This at least raises the possibility that these patients are more susceptible to disease recurrence and should be carefully monitored. Registry data are required to address this point.

Given the range of factors associated with response to immune checkpoint therapy in melanoma, albeit principally in the palliative setting, we specifically examined whether there was evidence that sex, BMI, immune-related adverse events, antibiotic use, or the neutrophil to lymphocyte ratio impacted upon disease recurrence during adjuvant treatment. Contrary to some reports, we found no evidence that females had a poorer response to adjuvant immunotherapy compared to males [32]. Similarly, we were unable to find evidence that an increased BMI was associated with a decreased risk of melanoma recurrence, given that the vast majority of patients with recurrence were overweight. In fact, only one of the patients who suffered from disease recurrence had a BMI < 24. It is worth bearing in mind that the presence of co-morbidities, including metabolic syndrome, fatty liver disease, heart disease, and type II diabetes may be associated with obesity and therefore modify the efficacy of immunotherapy rather than obesity influencing treatment response per se. Again, multi-national registry-based studies may help address this specific question. 

It has long been accepted that the discontinuation of immune checkpoint-based therapy due to immune-related adverse effects in melanoma is not associated with a poorer overall response to treatment. On the contrary, patients who develop immune-related adverse events often have a better overall response [36,37,38]. Our data are consistent with this finding, given that only two out of twelve patients developed immune-related adverse events in our cohort. In the retrospective analysis of Schadendorf et al. of pooled Phase II and III trials, almost 25% of patients discontinued combined immunotherapy (anti-PD-1 and anti-CTLA4) but nevertheless achieved a higher objective response rate than patients who did not discontinue therapy [38]. Of course, it should be borne in mind that combined immunotherapy does not currently play a role in the adjuvant setting. However, the development of immune-related adverse events may well paradoxically be reassuring to patients and clinicians that the adjuvant treatment is effective. 

In terms of other predictive biomarkers of treatment response, four of the patients who developed disease recurrence during adjuvant treatment had an elevated baseline neutrophil-to-lymphocyte ratio. Therefore, one-third of the cohort whose disease recurred were characterized by the presence of this negative prognostic factor [39]. Whilst our study was not powered or designed to specifically address the utility of baseline neutrophil-to-lymphocyte ratio measurement, our results suggest that this question should be addressed in prospective studies. Pending these results, clinicians would be well-advised to closely monitor patients with an elevated baseline neutrophil-to-lymphocyte ratio for disease recurrence.

Last but not least, none of our patients received antibiotic therapy immediately prior to immunotherapy, so we were unable to retrospectively examine the effect of antimicrobials on the efficacy of adjuvant immunotherapy. However, given evidence that the gastrointestinal microbiome modulated the efficacy of immunotherapy, clinicians should remain judicious in the use of antibiotics prior to and concurrently with immunotherapy [8,34,40,46,47]. Future research will also need to address the effect of tumor mutational burden, PD-L1 status, mismatch repair efficiency, and tumor-infiltrating lymphocytes, given that all these factors have been associated with response to immune checkpoint inhibitor-based therapy [54,55,56,57]

In conclusion, whilst adjuvant immunotherapy is now an established part of melanoma therapy, further studies and registry data are required to identify which patients are most likely to develop disease recurrence, at what point the risk is greatest, and the extent to which re-challenge with immunotherapy following surgical resection of metastases prolongs recurrence-free and overall survival in the real-world setting.

## 4. Materials and Methods

Following ethical approval from the University of Luebeck’s ethics committee (Reference number 22-001) and according to the Declaration of Helsinki principles, we retrospectively analyzed the electronic case records of patients who developed disease recurrence during adjuvant immunotherapy with either nivolumab or pembrolizumab between January 2018 and December 2021. Only patients who developed disease recurrence during the period (maximally one year) of adjuvant immunotherapy were included. Disease recurrence (loco-regional versus distant) was established radiologically according to RECIST criteria or histologically.

The following patient characteristics were recorded: sex (male/female), age (years), type of immunotherapy (nivolumab or pembrolizumab), mutation status (BRAF/NRAS/cKit), full blood count (eosinophil count and lymphocytes), baseline lactate dehydrogenase (LDH), and S-100 value at baseline; additionally, the points of progress were collated. Progression-free survival and overall survival were also calculated. In addition, we collated data on lymph node sonography, clinical and routine staging at the point of progress, and details of what treatment ensued. 

Descriptive statistics were performed using Microsoft Excel (version 2019). All statistical analyses were calculated using GraphPad Prism (version 8). *p* < 0.05 was considered statistically significant.

## Figures and Tables

**Table 1:** Overview of the patients.

Patient	Stage	Age	Therapy	Tumor Mutation	S-100 at Point of Recurrence	Treatment after Recurrence	Development of Further Recurrence?	Time to Further Recurrence
1	III C	63	Nivolumab	NRAS pq61R	not elevated	Continuation of immunotherapy	Yes (distant)	887
2	III B	67	Nivolumab	NRAS pq62R	not elevated	Discontinuation of immunotherapy	no	
3	III C	56	Pembrolizumab	NRAS pq61K	not elevated	Discontinuation of immunotherapy	no	
4	IV	53	Nivolumab	NRAS pq61K	not elevated	Discontinuation of immunotherapy	Yes (distant)	102
5	III B	57	Pembrolizumab	BRAV V 600 E	not elevated	Continuation of immunotherapy	no	
6	III B	53	Pembrolizumab	no mutation	not elevated	Continuation of immunotherapy	no	
7	III C	81	Pembrolizumab	NRAS pq61K	elevated	Discontinuation of immunotherapy	Yes (distant)	32
8	III C	71	Pembrolizumab	no mutation	not elevated	Discontinuation of immunotherapy	yes	
9	III C	52	Nivolumab	NRAS pq61K	not elevated	Discontinuation of immunotherapy	no	
10	III C	69	Nivolumab	NRAS pq61K	not elevated	Discontinuation of immunotherapy	yes (distant)	154
11	IV	61	Nivolumab	no mutation	not elevated	Discontinuation of immunotherapy	yes (distant)	122
12	IV	69	Nivolumab	NRAS pq61R	elevated	Discontinuation of immunotherapy	yes	30

**Table 2:** Biomarkers.

Patient	Sex	Melanoma Type	BMI	IRAE	Prior Use of Antibiotics	Neutrophil to Lymphocyte Ratio (Range: 0.78–3.53)
1	male	nodular	25.7	none	none	3.49
2	male	superficial spreading	31.4	none	none	3.88
3	male	superficial spreading	29.4	none	none	4.74
4	female	amelanotic	29.7	none	none	1.49
5	female	superficial spreading	24.8	none	none	1.81
6	female	nodular	27.9	none	none	2.71
7	female	acral	25.1	yes	none	2.58
8	female	acral	26.7	none	none	2.75
9	male	superficial spreading	26.5	none	none	2.25
10	male	superficial spreading	25.1	none	none	4.17
11	female	melanoma of unknown primary	36.8	none	none	2.86
12	male	superficial spreading	30.1	yes	none	3.83

## Data Availability

All data is available in the publication.
